# A Persistent Pneumonia or an Uncommon Condition: A Case of Immunotherapy-Induced Pneumonitis

**DOI:** 10.7759/cureus.104886

**Published:** 2026-03-09

**Authors:** Jessica Fidalgo, Carina Santos, Inês Sequeira, Catarina Valente, Patrícia Tenreiro

**Affiliations:** 1 Department of Internal Medicine, Guarda Local Health Unit, Guarda, PRT; 2 Department of Oncology, Guarda Local Health Unit, Guarda, PRT

**Keywords:** corticosteroid therapy, immune-mediated pneumonitis, immunotherapy, metastatic renal cell carcinoma, nivolumab

## Abstract

Immunotherapy with immune checkpoint inhibitors has revolutionized the treatment of metastatic renal cell carcinoma. However, it may be associated with severe toxicity, particularly immune-mediated pneumonitis. The present case report describes a 60-year-old patient diagnosed with stage IV clear cell renal carcinoma undergoing treatment with nivolumab, who developed severe partial respiratory failure refractory to empirical antibiotic therapy. After exclusion of other causes, namely, cardiovascular and infectious etiologies, immune-mediated pneumonitis was suspected, and systemic corticosteroid therapy was initiated, resulting in significant clinical and radiological improvement. This case illustrates immune-mediated pneumonitis in the context of nivolumab treatment, highlighting the importance of an accurate differential diagnosis in patients receiving immunotherapy and the early initiation of corticosteroid therapy when immune-related pneumonitis is suspected.

## Introduction

Immune checkpoint inhibitors such as nivolumab (anti-programmed death-1 (PD-1)) and ipilimumab (anti-cytotoxic T-lymphocyte-associated antigen-4 (CTLA-4)) have significantly improved survival outcomes and quality of life in patients with advanced renal cell carcinoma, becoming a cornerstone of systemic therapy in metastatic disease [1].

However, these agents are associated with a unique spectrum of immune-related adverse events resulting from nonspecific immune activation, which can affect virtually any organ system. Among these toxicities, immune-mediated pneumonitis represents an uncommon but potentially life-threatening complication, with an estimated incidence of 2-5% in patients receiving PD-1/PD-L1 inhibitor monotherapy and up to approximately 10% in combination regimens [2,3]. The clinical and radiological presentation is often nonspecific and may mimic infectious pneumonia, tumor progression, or other pulmonary conditions, particularly in patients with underlying pulmonary metastases. This overlap frequently leads to diagnostic delays and inappropriate initial management, increasing the risk of severe respiratory failure and morbidity.

Early recognition of immune-mediated pneumonitis is critical, as prompt initiation of systemic corticosteroid therapy is associated with rapid clinical improvement and favorable outcomes. However, delayed presentations and diagnostic challenges remain increasingly recognized in real-world clinical practice, particularly in patients receiving prolonged immunotherapy or maintenance regimens.

This case report aims to highlight the diagnostic challenges of immune checkpoint inhibitor-related pneumonitis in a patient with metastatic renal cell carcinoma initially misinterpreted as persistent infectious pneumonia, emphasizing how delayed recognition may lead to unnecessary antimicrobial therapy, clinical deterioration, and potentially severe respiratory failure and highlighting the importance of early suspicion and timely corticosteroid therapy.

## Case presentation

The patient is a 60-year-old man, previously independent in activities of daily living (Eastern Cooperative Oncology Group (ECOG) performance status 0), with a medical history of former smoking (20 pack-years), type 2 diabetes mellitus, arterial hypertension, dyslipidemia, sigmoid diverticulosis, and vitamin D deficiency.

**Figure 1:**
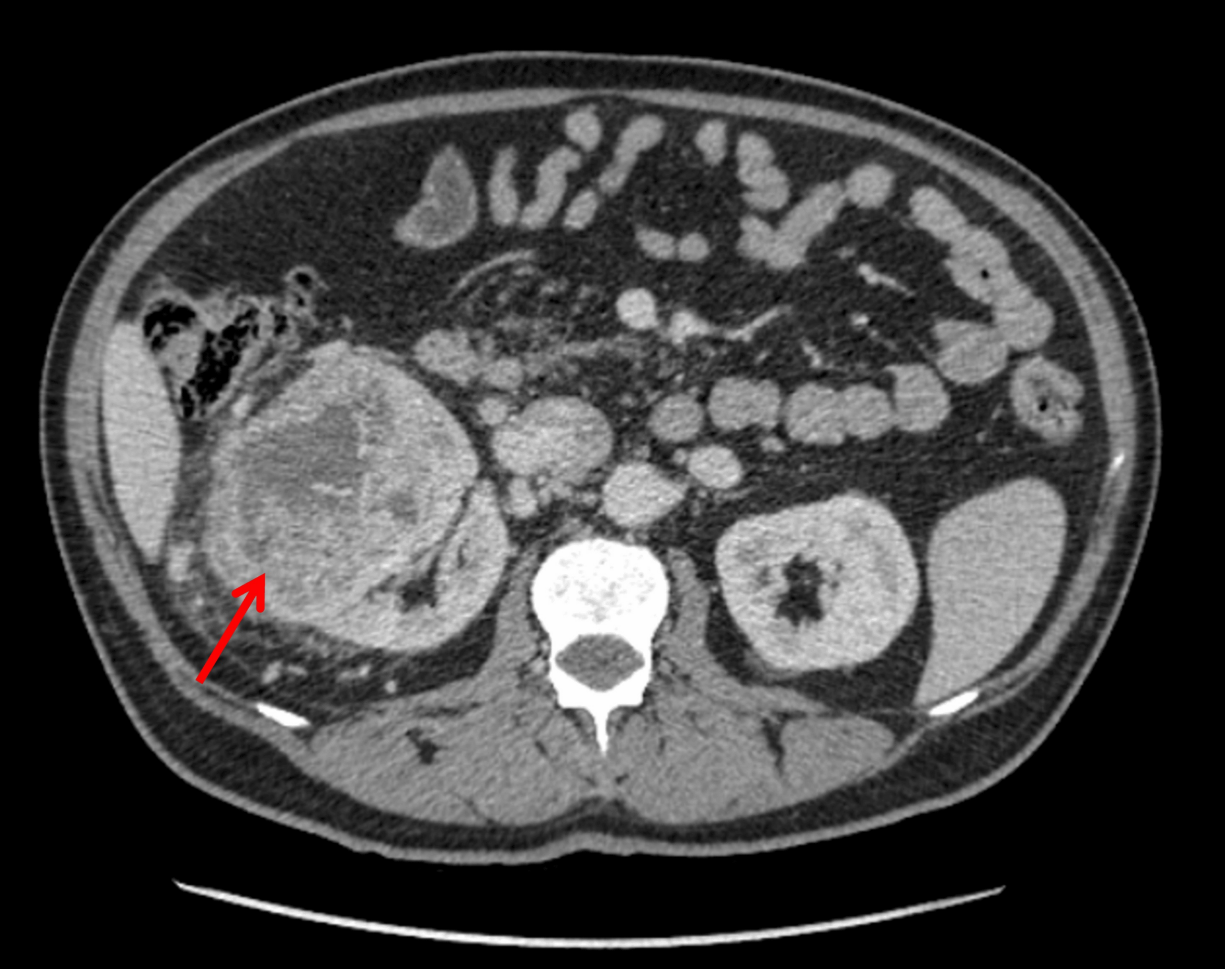
Abdominal CT (at initial diagnosis) Portal venous phase, axial view. CT showing a large heterogeneous right renal mass (135×115 mm). CT: computed tomography

**Figure 2:**
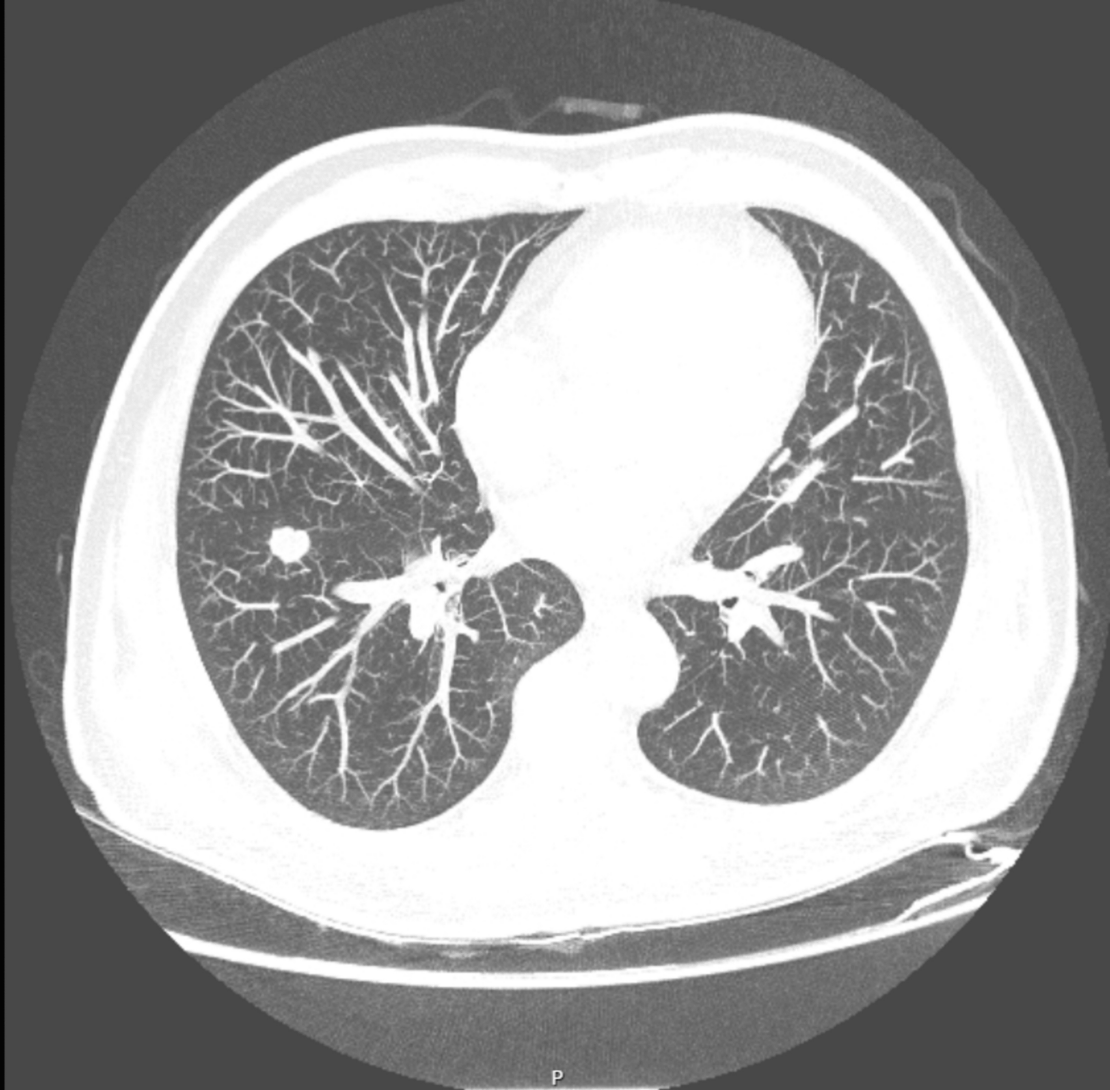
Chest CT (at initial diagnosis) Lung window, axial view. Initial presentation showing multiple pulmonary nodules consistent with metastatic disease. CT: computed tomography

At initial diagnosis, following renal ultrasound and abdominopelvic computed tomography (CT) performed due to right flank pain, gross hematuria, and urinary symptoms, a right renal neoplasm (Figure 1) with pulmonary metastases (Figure 2) was diagnosed. The patient underwent cytoreductive nephrectomy two months later. Histopathological examination confirmed clear cell renal cell carcinoma, International Society of Urological Pathology (ISUP) grade 2, with invasion of perirenal adipose tissue, classified as pT3a Nx M1, and an intermediate-risk International Metastatic Renal Cell Carcinoma Database Consortium (IMDC) score [4]. The patient was referred for follow-up in Palliative Care, alongside Oncology-Urology follow-up.

Three months after surgery, combined immunotherapy with nivolumab and ipilimumab was initiated as induction systemic therapy.

One month after starting immunotherapy, the patient developed a nonproductive cough, and chest radiography revealed a diffuse bilateral interstitial pattern increase, more pronounced in the middle and lower lung zones. Bronchodilator therapy was initiated empirically, and a chest CT scan was requested due to suspicion of disease progression or immune checkpoint inhibitor-related pneumonitis. Following the initiation of bronchodilator therapy, the patient experienced complete resolution of symptoms two weeks later. The complete resolution of symptoms following bronchodilator therapy, in the absence of corticosteroid treatment and without radiological progression, made immune checkpoint inhibitor-related pneumonitis less likely at this stage.

**Figure 3:**
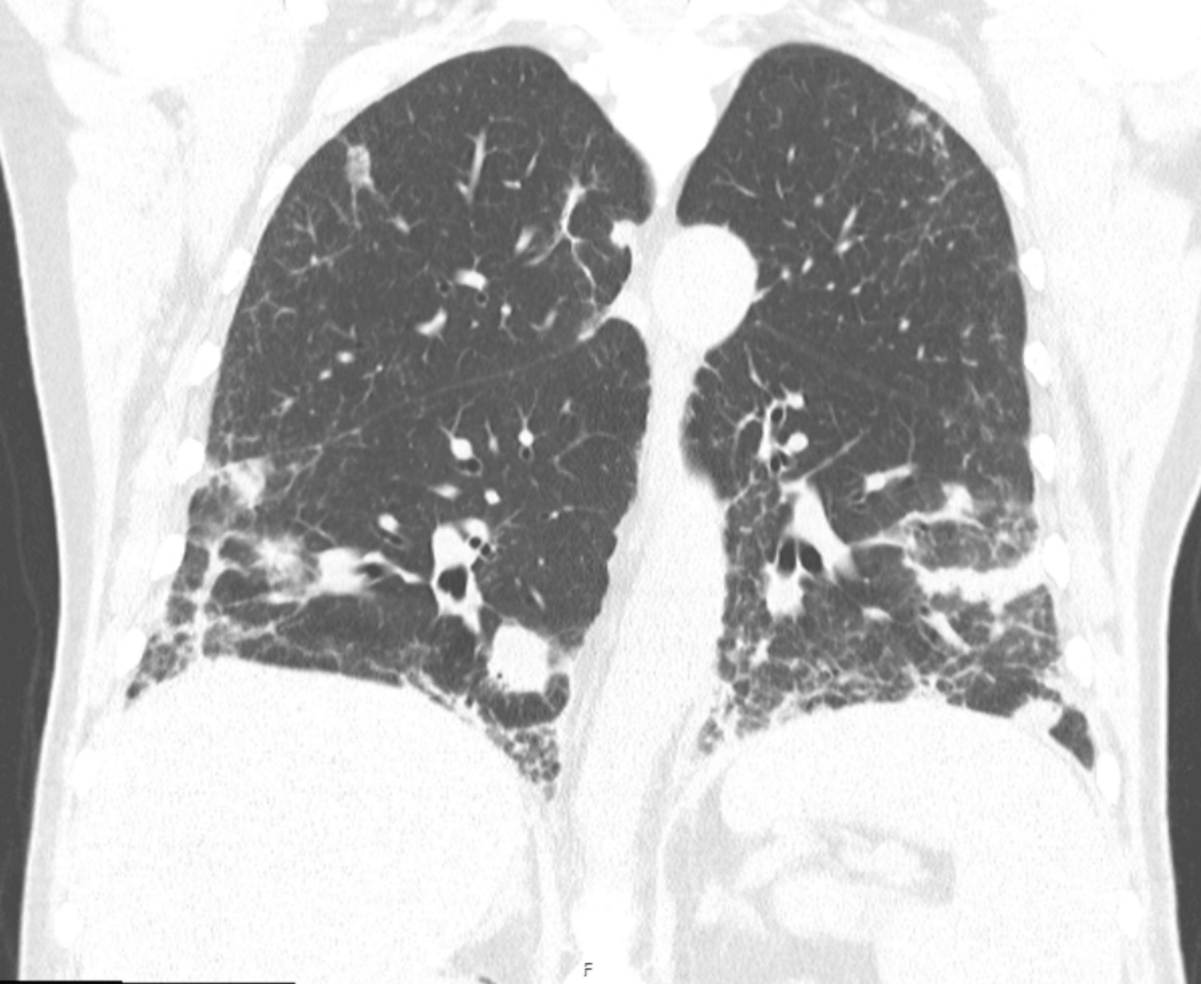
Chest CT (three-month follow-up after immunotherapy initiation) Lung window, coronal view. Multiple nodular lesions of varying sizes are seen scattered throughout both lungs, some of them cavitated, consistent with metastatic disease. There is also marked thickening of the interlobular septa, predominantly in the middle lobe and basal segments of the lower lobes, raising the possibility of carcinomatous lymphangitis. CT: computed tomography

The chest CT suggested the following: "multiple nodular lesions of varying sizes are seen scattered throughout both lungs, some of them cavitated, consistent with metastatic disease, showing an increase in both number and size compared to the previous examination. There is also marked thickening of the interlobular septa, predominantly in the middle lobe and basal segments of the lower lobes, raising the possibility of carcinomatous lymphangitis" (Figure 3).

In this context, second-line therapy with cabozantinib was proposed, while only nivolumab was maintained as maintenance monotherapy pending approval of the second-line agent.

**Figure 4:**
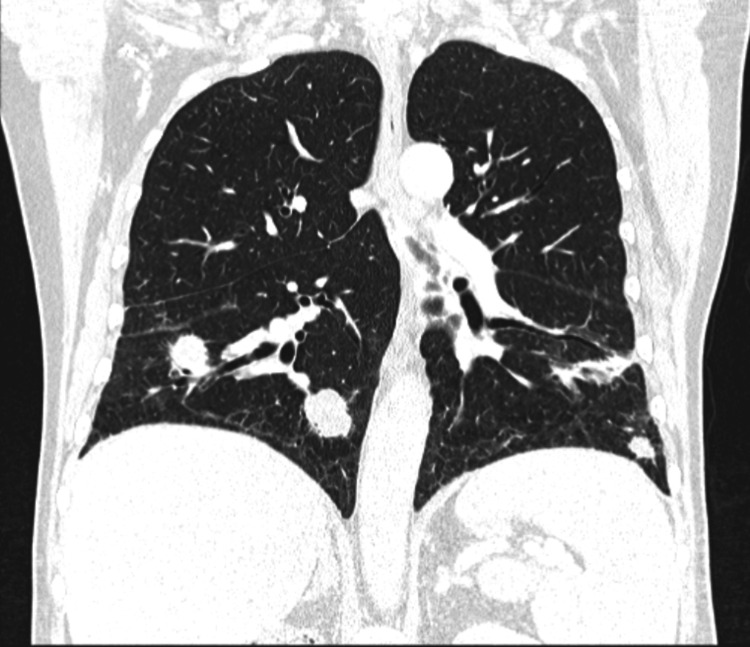
Chest CT (six-month follow-up after immunotherapy initiation) Lung window, coronal view. The largest nodules measured 23 mm (right lower lobe, unchanged), 16 mm (left lower lobe, unchanged), and 14 mm (lingula, previously 16 mm), indicating overall disease stability with reduction in selected lesions. A dimensional reduction of some nodules in the upper lobes and in the apical segment of the left lower lobe was observed. CT: computed tomography

Six months after immunotherapy initiation, and while on nivolumab monotherapy, chest CT at this stage demonstrated a reduction in the size and number of pulmonary nodules (Figure 4), consistent with a favorable treatment response. Given the absence of radiological progression suggestive of lymphangitic spread or clinical deterioration, nivolumab was continued.

**Figure 5:**
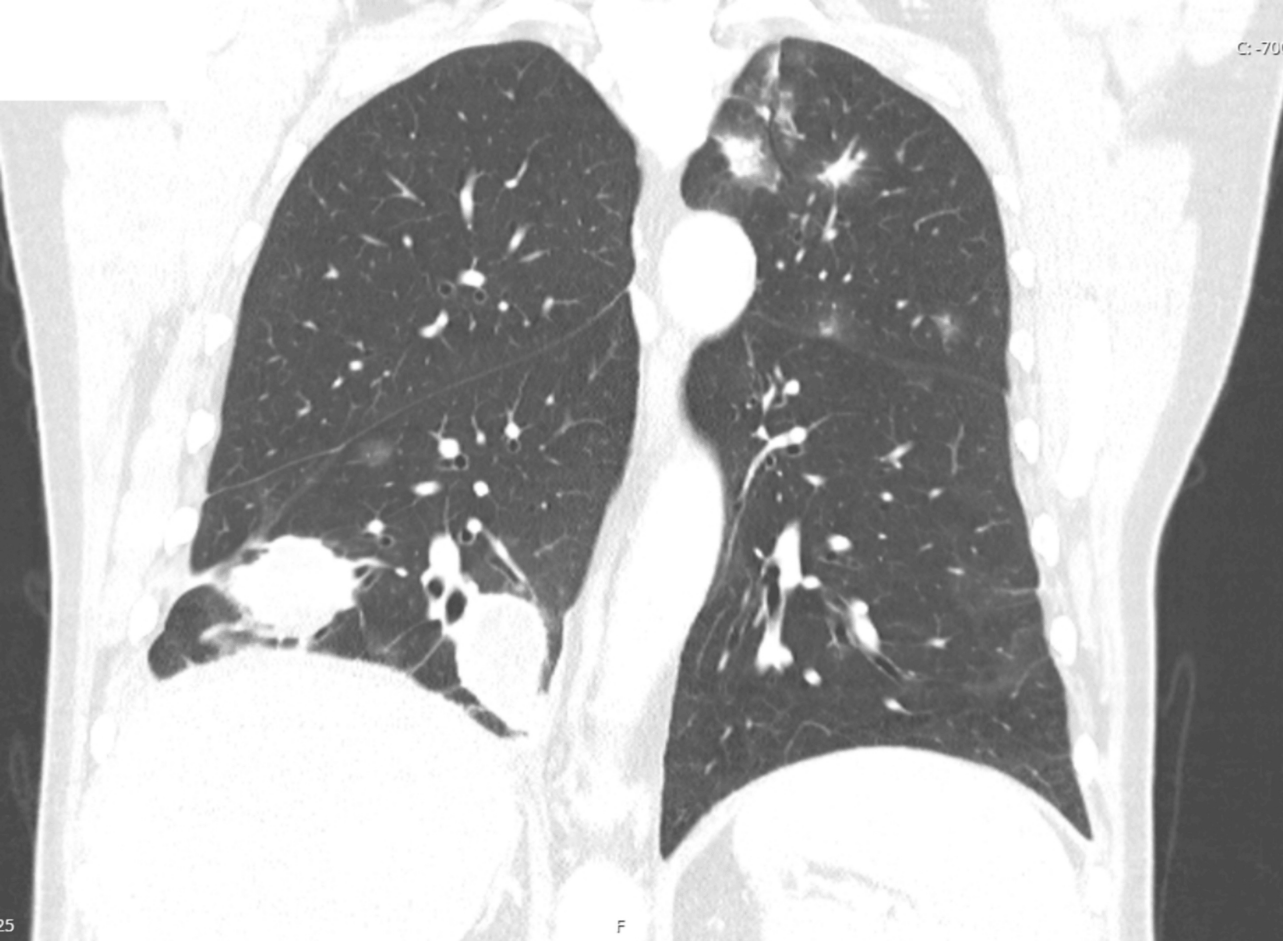
Chest CT (nine-month follow-up after immunotherapy initiation) Lung window, coronal view. Progression of pulmonary metastatic disease, with an increase in the size and number of bilateral pulmonary nodules: the largest lesions measure approximately 40 mm in maximum axial diameter in the right lower lobe (previously 23 mm), 21 mm in the left lower lobe (previously 16 mm), and 16 mm in the lingula (previously 14 mm); multiple new lesions are also observed, particularly in the upper lobes, the largest measuring 13 mm in the left upper lobe. CT: computed tomography

Nine months after immunotherapy initiation, progression of pulmonary disease was documented: "within the pulmonary parenchyma, multiple bilateral nodular lesions are identified, consistent with known pulmonary metastatic disease. Most demonstrate interval increase in size compared to the previous study, with an apparent increase in cystic component. The largest lesions measure approximately 40 mm in maximum axial diameter in the right lower lobe (previously 23 mm), 21 mm in the left lower lobe (previously 16 mm), and 16 mm in the lingula (previously 14 mm). Multiple new lesions are also observed, particularly in the upper lobes, the largest measuring 13 mm in the left upper lobe" (Figure 5). In the context of previously confirmed metastatic disease and radiological progression with multiple new bilateral pulmonary nodules, histological confirmation was not pursued. The imaging findings were considered highly suggestive of metastatic progression, and biopsy was deemed unlikely to modify the therapeutic strategy while carrying potential procedural risks.

**Figure 6:**
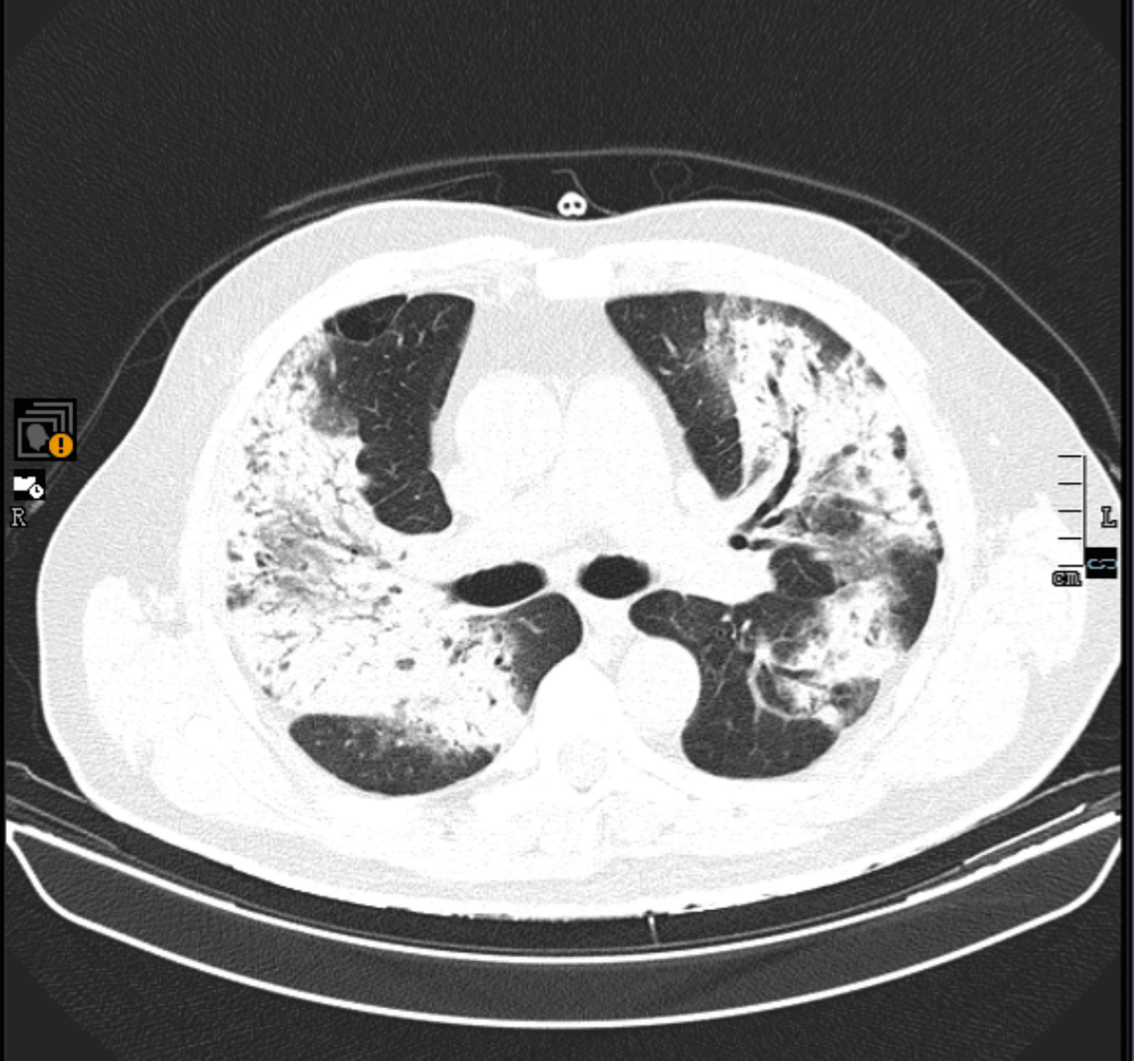
Chest CT (at admission to a hospital-at-home program) Lung window, axial view. CT showing parenchymal consolidations with air bronchograms scattered throughout the lung parenchyma, suggestive of a probable pneumonic process. Multiple associated bronchiectases are present, along with diffuse centrilobular and paraseptal emphysema. CT: computed tomography

Twelve months after immunotherapy initiation, the patient developed dyspnea on moderate exertion, consistent with presumed disease progression, and authorization was sought to initiate cabozantinib therapy, during which nivolumab monotherapy was continued. Dual bronchodilator therapy with budesonide and formoterol was initiated, with symptomatic improvement. One week later, the patient was evaluated in the Palliative Care clinic due to worsening dyspnea and nonproductive cough, associated with partial respiratory failure (PaO₂ 54.6 mmHg on FiO₂ 21%). Supplemental oxygen therapy at 1 L/min was initiated. He was reassessed again, and evaluation revealed further deterioration of respiratory failure, requiring an increase in oxygen supplementation to 4 L/min via nasal cannula (PaO₂ 59.7 mmHg on FiO₂ 21%). A chest CT (Figure 6) revealed the following: "multiple parenchymal consolidations with air bronchograms scattered throughout the lung parenchyma, particularly in the upper lobes and apical segments of the lower lobes, suggestive of a probable pneumonic process… multiple associated bronchiectases and diffuse centrilobular and paraseptal emphysema".

The presence of parenchymal consolidations with air bronchograms, associated pleural effusion, and the patient's underlying bronchiectasis favored an infectious etiology at initial presentation. Therefore, community-acquired pneumonia was considered the most likely diagnosis, and empirical broad-spectrum antibiotic therapy was initiated with ceftriaxone and azithromycin, with admission to a hospital-at-home program. The septic workup was negative (blood cultures, urinary antigen tests, and respiratory virus panel). The patient completed seven days of ceftriaxone and five days of azithromycin without clinical or laboratory improvement, with progressive worsening of respiratory failure, prompting transfer to an Internal Medicine ward. Empirical antibiotic therapy was escalated to piperacillin/tazobactam, and oxygen supplementation was increased to a Venturi mask with an FiO2 of 45%. A repeat septic workup remained negative.

**Figure 7:**
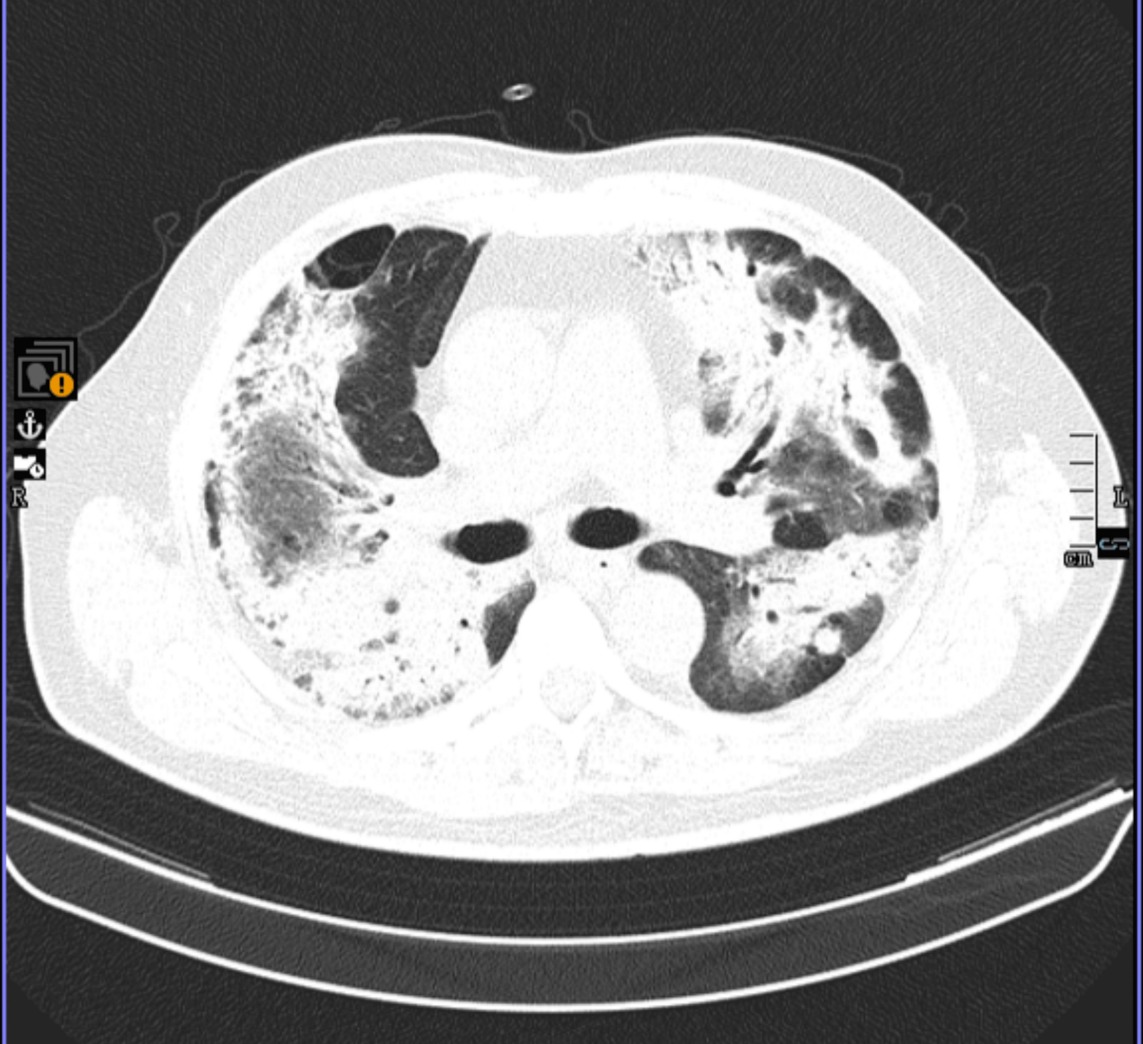
Chest CT (after 14 days of antibiotic therapy) Lung window, axial view. Persistence of bilateral pulmonary infiltrates without significant radiological improvement, reinforcing the differential diagnosis of immunotherapy-related toxicity. CT: computed tomography

After seven days of piperacillin/tazobactam, the patient continued to have severe partial respiratory failure, with harsh vesicular breath sounds and scattered bilateral crackles on lung auscultation, along with persistently elevated inflammatory markers (C-reactive protein (CRP) 8.77 mg/dL). Follow-up chest CT showed no improvement (Figure 7): "secondary nodular formations persist, as well as bilateral scattered infiltrates with the same topography, slightly denser compared to the previous examination, suggesting persistence of the inflammatory process".

**Figure 8:**
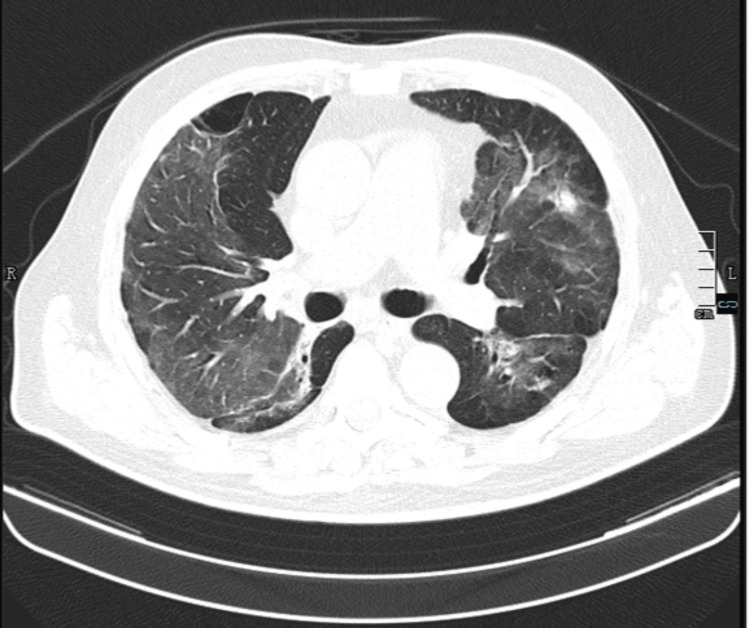
Chest CT (after 14 days of corticosteroid therapy) Lung window, axial view. CT demonstrating significant regression of bilateral infiltrates compared to the previous examination. CT: computed tomography

The absence of microbiological confirmation and lack of clinical improvement despite appropriate antibiotic escalation raised suspicion for alternative diagnoses, including immune checkpoint inhibitor-related pneumonitis. Bronchoscopy with bronchoalveolar lavage was considered as part of the diagnostic workup to further exclude infectious etiologies. However, due to the patient's significant respiratory compromise and increasing oxygen requirements, the procedure was considered high risk and was therefore not performed. Instead, the diagnostic approach relied on repeated microbiological investigations, radiological reassessment, and therapeutic response. A multidisciplinary discussion involving Internal Medicine, Oncology, and Radiology specialists was conducted during hospitalization after the absence of clinical improvement despite broad-spectrum antibiotic therapy. During this discussion, immune checkpoint inhibitor-related pneumonitis was considered the most likely diagnosis, and the decision was made to initiate intravenous dexamethasone at 15 mg/day, corresponding approximately to high-dose systemic corticosteroid therapy recommended for grade 3 immune checkpoint inhibitor-related pneumonitis. Based on the presence of symptomatic hypoxemia requiring supplemental oxygen and hospital admission, pneumonitis was graded as grade 3 according to the Common Terminology Criteria for Adverse Events (CTCAE) Version 5.0 [5]. Within 48-72 hours of corticosteroid therapy, marked clinical improvement was observed, including improved lung auscultation findings and oxygenation (with reduction of oxygen supplementation to 8 L/min), as well as a significant decrease in inflammatory markers (CRP 0.20 mg/dL). Although histological confirmation was not obtained, the diagnosis of immune checkpoint inhibitor-related pneumonitis was supported by the combination of clinical presentation, exclusion of infectious causes, compatible radiological findings, and the rapid and marked response to systemic corticosteroid therapy. After clinical stabilization and improvement in oxygenation, corticosteroid therapy was maintained for 14 days, followed by a gradual tapering regimen after hospital discharge in order to minimize the risk of relapse, in accordance with current guideline recommendations. After 14 days of corticosteroid therapy, follow-up chest CT (Figure 8) demonstrated bilateral infiltrates with significant improvement compared to the previous examination and complete resolution of the pleural effusion. 

The patient was discharged home with clinical stability and without the need for supplemental oxygen, on a corticosteroid-tapering regimen. He continued follow-up in Palliative Care and Urology/Oncology clinics. 

Although the previously identified pulmonary nodules remained dimensionally stable, new upper-lobe nodules emerged after nine months of treatment with nivolumab, raising concern for disease progression despite ongoing immunotherapy. Concurrently, the patient developed grade 3 immune checkpoint inhibitor-related pneumonitis requiring hospital admission and systemic corticosteroid therapy. According to current international guidelines, discontinuation of nivolumab is recommended in cases of grade 3 pneumonitis. Therefore, the decision to initiate cabozantinib reflected both the need to discontinue immunotherapy due to significant immune-related toxicity and the concern for emerging oncologic progression while ensuring continued systemic disease control.

The laboratory and gas exchange parameters during the patient's hospitalization are presented in Table 1.

**Table 1:** Laboratory and gas exchange parameters during hospitalization Serial laboratory values and arterial blood gas parameters obtained during hospitalization, illustrating persistent systemic inflammation and severe hypoxemia despite broad-spectrum antibiotic therapy, followed by rapid clinical, laboratory, and oxygenation improvement after the initiation of intravenous corticosteroid therapy. CRP: C-reactive protein; FiO₂: fraction of inspired oxygen; PaO₂: arterial partial pressure of oxygen; WBC: white blood cell count; PPT: piperacillin-tazobactam; Ster.: steroids

	Day 1 admission	Day 4	Day 7 D0 PPT	Day 10	Day 16 D1 Ster.	Day 17	Day 21	Day 30
WBC (5-10x10^3^/uL)	5.32	4.92	4.9	4.83	4.41	-	9.66	7.68
Neutrophils (1.87x10^3^/uL)	3.69	3.34	3.37	2.82	3.62	-	7.34	6.6
Hemoglobin (13.5-17.5 g/dL)	9.7	10.6	10.1	9.8	10.1	-	11.2	11.3
CRP (<0.50 mg/dL)	11.11	17.96	14.34	10.27	8.83	-	0.24	0.13
Procalcitonin (<0.50 ng/mL)	-	0.19	0.27	-	0.10	-	-	-
FiO2 (%)	28%	-	45%	45%	40%	38%	28%	21%
PaO2 (mmHg)	59.7	-	40.8	63.1	54	89.7	73.9	63.6
SatO2 (%)	90%	-	77.9%	92%	88.1%	98.1%	96.3%	92%

## Discussion

Immune checkpoint inhibitor-related pneumonitis is an uncommon but potentially life-threatening immune-related adverse event. Its reported incidence ranges from 2% to 5% in patients treated with PD-1/PD-L1 inhibitor monotherapy [2] and may reach approximately 10% in combination regimens, particularly with nivolumab and ipilimumab [6]. Although more frequently described early after treatment initiation, delayed presentations occurring months after exposure have been increasingly recognized [7]. This condition results from the increased activation of T lymphocytes and diffuse alveolar inflammation [7].

From a pathophysiological perspective, immune checkpoint inhibitor-related pneumonitis results from dysregulated T-cell activation and immune-mediated inflammation of the pulmonary interstitium, leading to lymphocytic infiltration, cytokine release, and diffuse alveolar injury. This mechanism differs fundamentally from infectious pneumonia, which is driven by microbial invasion and typically responds to antimicrobial therapy. The clinical presentation of immune-mediated pneumonitis is heterogeneous and nonspecific, commonly including dyspnea, nonproductive cough, and hypoxemia. Radiological findings are also variable, with ground-glass opacities, organizing pneumonia patterns, and interstitial infiltrates being the most frequently reported imaging manifestations [5]. This variability contributes to the diagnostic challenge, particularly in patients with underlying pulmonary metastases or prior radiological abnormalities, as illustrated in the present case.

In this patient, the diagnostic assessment was particularly challenging due to the coexistence of metastatic pulmonary disease and fluctuating radiological findings over time. The later development of diffuse bilateral parenchymal infiltrates occurred in the context of acute hypoxemic respiratory failure. Although histological confirmation was not obtained, lung biopsy was considered unlikely to alter clinical management in the context of advanced metastatic disease and significant respiratory compromise while also carrying procedural risks. Therefore, the diagnosis relied on a combination of clinical, radiological, and therapeutic response criteria.

Although an infectious etiology was initially considered and treated with appropriate broad-spectrum antibiotics, repeated microbiological investigations remained negative, and no clinical, laboratory, or radiological improvement was observed. At the time of hospital admission, the radiological findings of parenchymal consolidations with air bronchograms, combined with the presence of bronchiectasis and systemic inflammatory markers, strongly suggested community-acquired pneumonia, which initially justified empirical antibiotic therapy rather than immediate corticosteroid treatment. In retrospect, earlier consideration of immune checkpoint inhibitor-related pneumonitis might have allowed the earlier initiation of corticosteroid therapy. However, the initial clinical and radiological presentation strongly suggested an infectious etiology, supporting the initial empirical antibiotic approach. Furthermore, the absence of microbiological confirmation, the lack of response to two lines of broad-spectrum antibiotic therapy, and the rapid clinical and radiological improvement following corticosteroid initiation strongly supported an immune-mediated inflammatory process rather than infection.

Overall, the serial CT scans reflected two concurrent pulmonary processes: metastatic nodular disease exhibiting response and relative stability during treatment, followed by renewed oncologic activity (new nodules after nine months of nivolumab therapy), and a subsequent episode of grade 3 immune checkpoint inhibitor-related pneumonitis. The latter was diagnosed in the setting of acute hypoxemic respiratory failure with diffuse bilateral infiltrates, absence of response to antibiotic therapy, and rapid clinical and radiological improvement following systemic corticosteroid treatment.

According to current European Society for Medical Oncology (ESMO) and American Society of Clinical Oncology (ASCO) guidelines, the management of immune checkpoint inhibitor-related pneumonitis should be guided by severity grading based on clinical presentation and functional impact, using the Common Terminology Criteria for Adverse Events (CTCAE). Grade 1 pneumonitis is asymptomatic and identified only through clinical or radiological findings; grade 2 involves symptomatic disease requiring medical intervention and limiting instrumental activities of daily living; grade 3 is characterized by severe symptoms limiting self-care activities of daily living and typically requiring supplemental oxygen; grade 4 represents life-threatening respiratory compromise requiring urgent intervention; and grade 5 corresponds to death [5,8-10].

In grade 1 pneumonitis (asymptomatic, involving <25% of lung parenchyma), close clinical monitoring is recommended, including pulse oximetry and chest CT imaging. Immunotherapy may be temporarily withheld, and patients should be reassessed every 2-3 days. If symptoms worsen, management should escalate according to higher grades [9].

In grade 2 pneumonitis (new or worsening respiratory symptoms such as dyspnea or cough, with increased oxygen requirement), immunotherapy should be withheld, and patients should undergo a thorough infectious workup, including sputum, blood, and urine cultures, with consideration of bronchoscopy and bronchoalveolar lavage if clinically indicated. Oral corticosteroids (prednisone 1 mg/kg/day or equivalent) should be initiated, with escalation to intravenous corticosteroids if no improvement is observed within 48 hours [9].

In grade 3 or 4 pneumonitis (severe symptoms, hypoxemia, life-threatening respiratory compromise, or acute respiratory distress syndrome (ARDS)), permanent discontinuation of immunotherapy is indicated. Hospital admission is required, along with high-dose intravenous methylprednisolone (1-2 mg/kg/day), empiric antibiotic coverage, and multidisciplinary evaluation. If there is no improvement within 48-72 hours, additional immunosuppressive therapies such as infliximab, tocilizumab, mycophenolate mofetil, intravenous immunoglobulin, or cyclophosphamide may be considered. Corticosteroids should be gradually tapered after clinical improvement to grade <1, typically over 4-6 weeks for grade 2 and ≥6-8 weeks for grade ≥3 pneumonitis [9].

Regarding re-challenge with immune checkpoint inhibitors after pneumonitis, current guidelines generally recommend permanent discontinuation in patients with grade 3 or 4 pneumonitis due to the risk of recurrence. In selected cases of grade 1 or resolved grade 2 pneumonitis, cautious reintroduction may be considered under close clinical monitoring.

This case highlights several clinically relevant aspects. In patients receiving immune checkpoint inhibitors who present with respiratory symptoms, the diagnostic approach must include a broad differential diagnosis, such as infectious pneumonia (typical and opportunistic), tumor progression, carcinomatous lymphangitis, pulmonary embolism, and cardiac-related pulmonary edema. Immune-mediated pneumonitis should remain a key consideration within this framework, particularly when microbiological investigations are negative and clinical deterioration persists despite appropriate antimicrobial therapy.

Additionally, delayed-onset pneumonitis may occur after prolonged exposure to immunotherapy, including during maintenance monotherapy. Early recognition, multidisciplinary evaluation, and timely initiation of corticosteroid therapy are essential to prevent progression to severe respiratory failure and to improve clinical outcomes.

Similar cases of delayed immune checkpoint inhibitor-related pneumonitis have been described in patients receiving nivolumab and other PD-1 inhibitors, including those treated for metastatic renal cell carcinoma. These reports highlight that pneumonitis may occur several months after treatment initiation and frequently mimics infectious pneumonia, creating significant diagnostic challenges in clinical practice. Radiological findings often overlap with infectious or inflammatory patterns, including ground-glass opacities, organizing pneumonia, or bilateral consolidations. In many cases, as observed in our patient, the absence of microbiological confirmation and the rapid clinical and radiological response to systemic corticosteroid therapy are key elements supporting the diagnosis of immune-related pneumonitis [11-13].

The coexistence of metastatic pulmonary disease and subsequent inflammatory infiltrates created a particularly complex diagnostic scenario in which tumor progression, infection, and immune-related toxicity overlapped. This overlap reinforces the importance of multidisciplinary collaboration and systematic reassessment in patients receiving immunotherapy who develop respiratory deterioration.

Finally, this report illustrates the diagnostic challenges frequently encountered in real-world clinical practice. In retrospect, earlier consideration of immune checkpoint inhibitor-related pneumonitis might have allowed the earlier initiation of corticosteroid therapy. However, the initial clinical presentation and radiological findings strongly suggested an infectious etiology. Additionally, the absence of histological confirmation represents a limitation of this report, although the diagnosis was supported by clinical evolution, exclusion of infection, and the rapid response to corticosteroid therapy.

## Conclusions

Immunotherapy-associated pneumonitis, although uncommon, should be considered in the differential diagnosis of patients receiving immune checkpoint inhibitors who present with new-onset respiratory symptoms or hypoxemia, particularly when microbiological investigations remain negative. As illustrated in this case, distinguishing immune-mediated pneumonitis from infectious pneumonia or tumor progression may be particularly challenging in patients with underlying pulmonary metastases and overlapping radiological findings.

This report highlights the importance of dynamic clinical reassessment, multidisciplinary collaboration, and systematic exclusion of alternative diagnoses. Early recognition and prompt initiation of systemic corticosteroid therapy remain essential to prevent progression to severe respiratory failure and to improve clinical outcomes.
